# Supplementary material for: Are carbon emissions trading and green financial instruments synergistic? -Comprehensive quantitative research based on content analysis
Source: PLoS One. 2024 Mar 7;19(3):e0298601. doi: 10.1371/journal.pone.0298601 (PMC10919601; doi:10.1371/journal.pone.0298601)
Supplement: S1 Data — (ZIP) [file pone.0298601.s001.zip › Data/地级市Z_F工作报告.zip!/╕ú╜¿/╒─╓▌╩╨/└┤╫╘╣1⁄2╓┌║┼í╛┬φ┐╦╩2╛▌═°í┐_2005.docx]

各位代表：

现在，我代表漳州市人民政府向大会作政府工作报告，请予审议，并请各位政协委员和其他列席人员提出意见。

一、2004年政府工作回顾

2004年，我市各级政府在省委、省政府和市委的正确领导下，以邓小平理论和“三个代表”重要思想为指导，认真贯彻党的十六大和十六届三中、四中全会精神，牢固树立和落实科学发展观，按照市委确定的积极参与海峡西岸经济区建设、打造“三个基地”、构建生态工贸港口城市的总体部署，扎实推进各项工作，超额或基本完成了市十三届人大五次会议确定的各项目标任务。

（一）经济运行呈现近些年来较为可喜的新的态势

完成地区生产总值702亿元，增长11.6%（与上年度比较，下同）；农业总产值246亿元，增长5%；工业总产值817亿元，增长23.9%，规模工业总产值465亿元，增长44.1%；全社会固定资产投资163亿元，增长15.5%，其中城镇以上固定资产投资136亿元，增长17.8%；全社会消费品零售总额199亿元，增长10.8%；外贸出口21.56亿美元，增长76.3%；财政总收入（同口径）40.13亿元，增长29.6%，地方级财政收入（同口径）22.25亿元，增长29.0%；城镇居民人均可支配收入10117元，增长11.8%，农民人均纯收入4320元，增长8.5%。上述指标中，地区生产总值、工业总产值、规模工业产值、固定资产投资、外贸出口总额、财政收入、农民人均纯收入增加额度为近些年来最大，增幅为1999年来最高；规模工业产值增幅、财政总收入和地方级财政收入（同口径）增幅为全省第一，外贸出口总额增幅为全省第二。

（二）工业立市战略带动增强综合实力凸显新的动力

工业发展带动经济结构优化效益提升。按照“4+3”产业（食品、机械、材料、能源4个主导产业和电子、医药、家俱3个重点产业）布局，以园区为载体、大中项目为重点，国有民营外资多元推动，促进产业聚集，既壮大了工业群体，又带动了三次产业协调发展。规模工业企业首次突破1000家，主导产业、重点产业产值占规模工业比重上升到85.7%。工业增加值249亿元，增长22.5%，对GDP增长贡献率达62.4%，三次产业结构比例由21∶39.4∶39.6 调整为20.3∶41.8∶37.9。规模工业综合效益指数为145.7，同比提高13.3个百分点，工业税收达20.36亿元，增长49%，对财政总收入增长贡献率达73.1%。

工业立市拉动农业产业化提升水平，促进农民增收。食品工业发展带动农产品加工出口率增长6个百分点。以“4142”基地为重点，大力实施绿色行动计划，新增农业产业化龙头企业生产基地15万亩，新增无公害农产品生产基地10万亩、绿色食品基地5万亩；新增规模食品工业企业11家、外资农业企业72家、新型农业合作组织24家，紫山集团被列为国家级重点龙头企业和全国优秀龙头食品企业；新增绿色食品标志25个，11个产品获得AA级“绿标”和有机食品标志使用权。二、三产业的发展为农村劳动力转移拓展了新的空间，全年转移9.49万人，农民人均收入增加额中劳务收入达60%。地方财政收入的增加增强了支持农业的财力，全市农业投入增长14.2%。

工业立市推动县域经济总量扩张财力增加。各县（市）GDP增额为76亿元，总量达548亿元，占全市比重达78.1%；财政总收入、地方级财政收入增额分别为4.87亿元、1.85亿元，总量分别为22.49亿元、10.69亿元，占全市总量比重分别由56.9%、51.3%提升为59.2 %、53.2%；财政总收入龙海市突破10亿元，芗城区突破5亿元，漳浦突破2亿元；漳州开发区和蓝田、龙池、角美、金峰开发区财税总收入均超亿元。各县（市、区）在比学赶超活动中敢于比先进、找差距、争位次，龙海、芗城展现了率先崛起的良好势头。

（三）拉动经济成长的主要因素显现新的活力

国有企业体制改革展现新效益。列入全省战略性结构调整的27个项目改制成功，18家市直国有工贸企业完成改制；国企整体活力增强，总税收增长16.07%，纳入快报的83家国企扭亏幅度达41%。龙溪轴承集团等国有企业运用市场机制，依靠科技创新，增强了核心竞争力，产值、创汇、税利大幅上升；“漳州发展”等上市公司发挥融资平台作用，有力支持市政基础设施建设。

激活民营经济的措施初显新成效。民营投资起步态势良好，城镇以上民间投资增长67.6%；投资500万元以上工业项目民营占项目数61%，占投资额53.9%；在建工业项目民营占项目数62.7%，占投资额62.8%。民营企业群体逐步成长，新增规模企业民营占73.6%，民营规模工业产值增长41.7%，出口创汇增长127%；民营企业税收增长26.45%。

外经外贸在经济运行中继续发挥重要支撑作用。精心组织参与“6·18”全省项目成果交易会、“9·8”国际投资洽谈会，成功举办“4·9”漳台经贸恳谈会和“11·28”海峡两岸花博会，各项展事活动成果好于往年，尤其是第六届花博会升格为全国性以农业为主的专业商展活动并向其他行业拓展。全市新批外资项目269个，与前年基本持平，合同利用外资5.63亿美元，实际到资5.31亿美元，分别增长10.9%和15.3%。外贸出口额突破20亿美元大关。外资企业税收总额13.95亿元，增长46.01%，比重达39.25%。

固定资产投资初显新规模。全社会固定资产投资自2001年以来增幅首次突破二位数。全市66个重点项目全面完成年度投资计划，漳龙高速公路高质量通过验收，提前九个月全线通车；后石电厂6号机组、郭坑大桥、医护高专一期、聚善堂药业配送中心等一批重点项目建成，漳州港20万吨级矿建泊位和15万吨级油码头建设正式启动。

市场体系建设和服务行业发展初显新势头。新建6家、改造26家生鲜超市、连锁商场，新引进上海“好又多”和福州环宇等2家百货企业，改造浦口粮食、闽南商业、浦东副食品等3家专业批发市场，漳州开发区粮食仓储被定点为国家级粮食物流港口节点，吉马物流一期主体工程封顶。全市金融机构存款余额369.86亿元，比年初增加54.55亿元；贷款余额258.22亿元，比年初增加21.32亿元；城乡居民储蓄达249.14亿元，比年初增加35.39亿元。房地产业投资增长55.9%；旅游行业总收入增长12.5%，接待国内外游客增长11%；汽车销售业总收入增长20.4%；港口吞吐量增长40.15%，达1520万吨。

（四）社会事业协调发展取得新的成果

科技教育事业进步较为明显。全市高新技术企业产值突破100亿元，增长60.2%；新增国家级火炬项目7个，其中3个获优秀火炬项目；11家企业获“省级高新技术企业”称号，9项成果获省级科技进步奖；芗城区、龙海市获全国性科技工作先进集体称号。继续巩固九年义务教育，农村中小学危房改造完成110.3%；高中阶段教育当年适龄人口入学率达60.6%，毛入学率达50.6%，分别提高11和15个百分点；高校园区初成规模，少体校投入使用，卫校升格为医学护理职业学院并迁入新校区，职业技术学院通过教育部人才培养工作评估。

文化体育卫生事业取得新发展。实现广播电视综合业务网络集成化。整治规范文化市场，繁荣文化创作，市木偶剧团获国际木偶节最高奖，市区香港路、台湾路街区获联合国亚太地区文化遗产保护奖。龙海市榜山镇、龙文区步文镇、芗城区南坑街道办事处获全国性体育先进集体称号，诏安吴燕聪夺得雅典残奥会跳高冠军，华阳电业公司捐建华阳体育馆进展顺利。城镇医疗卫生体制改革取得进展，疾病预防控制体系和医疗救治体系部分项目投入使用。

贯彻三项基本国策全面完成责任目标。人口自然增长率为6.0‰，实现了控制目标；龙海市创建全国计生优质服务先进市通过验收，芗城、长泰、南靖获省计生优质服务县（区）称号，漳浦县村级计生管理员制度改革经验在全省推广。扎实推进开发区整合工作，将原有各类开发区重组为8个开发区；完成土地整理1.3万亩，连续6年实现耕地占补平衡，龙海市国土局获全国国土资源管理先进单位称号。创建国家环保模范城市深入推进，重点流域水环境、市区三湘江综合治理及烟尘污染等专项整治取得扎实成效，长泰县通过国家级生态示范县验收。

同时，全市老年、残联、妇女儿童、外事侨务、民族宗教、社科、人防、消防、地震、气象、档案、方志等工作都取得了可喜的新成绩。

（五）构建和谐社会的工作成效跃上新的层面

精神文明建设活动进一步深化。以“重信誉、守信用、讲信义”评议活动为载体推动“信用漳州”建设；创建全国文明城市活动全面展开，在基层多行业、多层面广泛开展创建“文明单位”活动，夯实了文明城市创建活动的社会基础。以学习、实施《行政许可法》为契机，深入推进依法治市进程，执法机关、行政部门依法行政自觉程度和工作水平有了新的提升。市公安局戒毒所获全国“人民满意公务员集体”称号。

努力改善城镇生活环境。中心市区建成区面积扩展。江滨路全线启动，龙文南路、西洋坪路竣工，新客运站主楼等一批重点项目投入使用。市区生活垃圾实现集中无害化处理，污水管网铺设完成年度计划；新增公共绿地114公顷，人均达7.1平方米；华元小区获国家鲁班奖。开展创建全国卫生城市活动，市区及南靖、长泰通过省级复核验收，龙海、诏安获得省级卫生城市（县城）命名。

重点解决农村生产生活突出问题。建设农村等级公路1116公里，完成年度计划109%。农村“六千”水利工程完成年度改造建设计划。创新贫困村帮扶机制，加强扶贫挂钩制度，对17个挂钩重点村筹集资金716万元，扶持以公路为重点的89个项目建设；实施“造福工程”，完成1190名贫困户的异地搬迁；有效改善了老、少、边、岛等贫困地区农村的发展条件。集体林权制度改革进展顺利，林权证发放率居全省首位；集体土地使用权流转制度平稳实施。

切实加强城乡居民生活保障。新增就业岗位4.27万个，下岗人员再就业5544人，城镇居民登记失业率为4.2%，南靖县劳动保障就业中心被评为全国就业先进单位。企业退休人员基本养老金和失业人员保险金实现100%社会化足额发放。城乡居民最低生活保障实行了统一发放制度，落实到户到人。农民工工资清欠工作取得明显成效。农村“减负”措施进一步落实，人均“减负率”达94.1%。扎实为群众办好事、做实事，市委市政府确定的15件为民办实事项目件件落实。

全面启动“平安漳州”建设。认真落实社会治安综合治理责任制。积极办理群众来信来访，有效防止、化解群体性事件；强化“严打”，加强基层防范体系建设，社会治安进一步好转，群众满意率提升。严格安全生产责任管理，重大责任事故得到有效遏制。进一步发挥工商、税务、质监、药监、公安、统计、审计、物价等执法部门作用，重点治理制假售假等侵害消费者利益现象，扩大了市场秩序整规成果。

进一步推进民主法制建设。认真执行人民代表大会决议决定，妥善办理人大代表建议、政协委员提案。加强基层政权和社区建设，提高村（居）民民主自治能力。继续扩大政务公开范围，自觉接受社会各界法律监督、民主监督。把国防动员工作列为政治建设重要内容，民兵预备役工作取得新成果，组建民兵船运团，成功组织“过得去”演练。政府机关廉政建设进一步加强，领导干部廉洁自律和廉政责任制得到较好落实。

（六）全市人民创新机制、推动发展焕发新的风貌

去年，我市在发展中面临突出的困难和挑战。全市干部群众在市委坚强领导下，主攻突破点，狠抓薄弱点，培育增长点，坚持以发展为第一要务，坚持以项目为着力点，坚持从体制改革入手，寻求破解难题的思路、方式和措施。实施新一轮行政审批制度改革，取消和调整市级审批事项17项，扩大县级事权115项，县域经济管理权限进一步放活。金融、工商、税务、海关、检验检疫等部门在严格执法的同时，改进机制，为化解经济发展的制约因素提供了新的保障。同时，以盘活土地资源，用地与效益挂钩；用活融资政策，激活民间资本；加强电力调度，补助企业自备电源；加强劳务培训，组织外出招工等措施，较为有效地破解了用地、融资、电力、用工等方面的难题，促进经济链条的良性运转。全市人民面对挑战敢为有为，战胜了比预料更为严峻的困难，取得了比预料更为良好的业绩。

各位代表！过去的一年，我们所取得的成绩来之不易。这是省委、省政府和市委正确领导的结果，是市人大依法监督、市政协积极参政议政的结果，是全市人民和外来劳动者以及各方面大力支持的结果。借此机会，我代表漳州市人民政府向全市人民，向驻漳的中央和省属单位，向驻漳人民解放军和武警官兵，向各民主党派、人民团体，向所有关心、支持漳州建设和发展的各界人士、港澳台同胞和海外侨胞致以崇高的敬意和衷心的感谢！

去年，我市经济社会发展展示了新的良好态势，但仍然存在不少困难和问题，工作中也存在许多薄弱环节：经济总量偏小，经济增长的速度与周边先进地区相比有较大差距，经济总体质量不高；县域经济发展不平衡，一些地方公共财力薄弱，财政紧张状况在短期内难以得到有效缓解；项目工作同发展态势不相适应，尤其缺乏骨干支撑项目、大型生产性项目，投资增长后劲较弱，个别项目进展缓慢；民间投资自主动力不足，产品、品牌、产业缺乏整体性竞争力，市场体系建设的推进机制不够有力，与产业发展的互动效应不够明显；农业基础薄弱，抵御灾害能力不强，农民增收缓慢；一些体制性障碍还没有得到根本性解决，企业、群众对办事难、审批难的问题仍有不少反映，社会治安仍有突出问题，投资软环境有待进一步改善。对此，各级政府将继续予以高度重视，采取得力措施，有效解决这些前进中的困难和矛盾。

二、2005年政府工作主要任务

2005年是我市全力打造三个基地、发展港口经济的全面启动之年，是漳州撤地建市20周年的喜庆之年，也是完成“十五”计划、夯实“十一五”发展基础的关键之年。我们必须站在新的起点，瞄准新的目标，采取新的举措，开创新的局面，推进新的跨越。今年政府工作的总体要求是：以邓小平理论和“三个代表”重要思想为指导，认真贯彻党的十六大和十六届三中、四中全会精神，树立和落实科学发展观，积极参与海峡西岸经济区建设，围绕打造三个基地、建设生态工贸港口城市的目标定位，以工业立市为主导，以培育临港产业、壮大县域经济、实施项目带动、激发民营活力、增强财政实力、统筹协调发展为重点，全力推进经济建设和各项事业再上新台阶。今年国民经济和社会发展主要预期目标是：GDP增长11%，其中一、二、三产业增加值分别增长4%、15%和10.5%；全社会固定资产投资增长12%；财政总收入同口径增长20%，其中地方级财政收入同口径增长14.8%；社会消费品零售总额增长11%；外贸出口增长20%；实际利用外资增长15%；城镇居民人均可支配收入增长8%；农民人均纯收入增长5%；居民消费价格总水平上升控制在4%左右；城镇登记失业率控制在4.3%以内；人口自然增长率控制在7‰以内。

实现今年各项目标任务，重点抓好以下工作：

（一）培育港口经济，打造三个基地

积极参与海峡西岸经济区建设。建设海峡西岸经济区的构想有利于推进我市在更高的层面、更宽的平台上拓展发展空间。我市要围绕打造三个基地、构建生态工贸港口城市的目标，按照全省关于突出发展、突出对台、突出统筹的指导原则和构建九大支撑体系的总体部署，依托港口、对台、农业三大优势，努力构建具有优势的特色产业体系、突出对台的开放型流通体系、适度超前的基础设施体系、联动共荣的港口城镇体系、协调进步的科教文化体系和永续发展的生态环境体系。主动融入厦门湾经济集群，以建设厦门湾组合港为启动点，积极推动构建城市联盟体，对接泉厦、闽西、粤赣等区域开展经贸协作，促进联动发展。

努力构建港口经济的基本框架。围绕把厦门湾建成我国沿海主枢纽港、集装箱运输干线港和区域性航运物流中心的整体目标，加快我市港口建设总体规划修编，完善相关专项规划编制。以招银港区、古雷港区为重点，引进多元投资主体，加快港口开发建设。重点发展港口运输产业，积极扶持船舶修造业、海洋运输业及船务、联运等相关服务行业，拓宽货流畅通渠道，扩大港口吞吐能力；努力扩展集装箱班轮航运，争取开辟对台直航航线，创造条件建成国际航线补给基地。大力发展港口物流业，拓展港口服务区建设，培育港口物流龙头企业，以木材、钢材、建材、化工材料为重点，建设大规模专业储运基地，发展仓储、加工、包装、配送及信息等多功能物流服务。突出发展临港工业，充分利用港口腹地，加快漳州开发区等临港工业区开发建设，引进、培育石化、机械、材料、电子等产业大中型项目，带动临港工业的整体性开发和产业聚集。

推进三个基地建设全面启动。发挥我市对台经贸合作的比较优势，以引进加工制造业为主，全方位拓展合作领域，构建台商投资密集的两岸经贸合作基地；发挥我市港口等工业资源的相对优势，以培育临港工业集群为主，提升传统产业，扶持高新技术产业，构建产业关联紧密的新兴制造业基地；发挥我市农业的综合优势，以扩大绿色、有机食品的种养、加工为主，促进食品产业迈向专业化、特色化、规模化、标准化生产的轨道，构建适应多元市场的优质食品供应基地。根据市委关于打造三个基地的部署要求，确定发展目标，编制建设规划，选定重点项目，建立责任机制，推动三个基地建设取得扎实成果。

（二）坚持工业立市，推进产业聚集

依托优势产业，扩展产业群体。以“4+3”工业产业体系为支撑，重点培育汽车船舶、港口设备、机械配件、家用电器、电子仪表、石英钟表、罐头食品、粮油加工、水产加工、建材、医药、家俱等12个产业集群，重点扶持60个省级工业增长点、47家市级重点企业和278个新增产值1000万元以上项目，引导向规模化、产业化、集约化方向发展；加快575个投资500万元以上工业项目建设，重点推动31个投资上亿元的重点工业项目加快建设进程，争取有三分之一以上竣工投产，努力实现新增规模工业企业100家以上，新增一批年销售收入超亿元企业，规模工业产值超600亿元，全部工业总产值突破1000亿元大关。

对接宏观导向，壮大产业基础。根据国家、全省产业发展布局，完善产业发展规划。着眼于周边地区产业发展动态，加强产业对接，主动承接厦门汽车、电脑、电视、手机等整机组装产业向外转移的零部件生产项目，积极承接泉州、汕头等地向外扩散的服装、制鞋、玩具、石材、化工等大量性用工、用电、用地的产业项目。以重点工业企业、成长型企业为基础，注重引办产业链重点环节项目和填补空白项目，促进产业配套。以骨干企业为龙头，以优质项目为核心，以股份合作为纽带，引导多元投资主体实行强强联合，组成团队投资大中型项目，培育强势企业、优质品牌和特色行业。

推进技术创新，培植高新产业。以发展新型金属、化工材料为重点，培育新材料产业集群；建设科明光电照明产业园，规划开发模具工业园。以科技孵化基地为基本载体，构建科技基础平台、创业服务平台和科技成果转化平台；进一步完善以提高企业自主开发能力为主的科技创新机制，培育一批拥有自主知识产权的高新技术产品，争取新发展省级高新技术企业10家以上。以政府信息工程推进企业信息化进程，重点抓好龙轴等3个省级和10个市级示范企业的信息化建设，带动一批企业信息化改造取得重大突破。

（三）提高农业综合生产能力，促进农民增收

“三农”工作必须以贯彻落实中央一号文件精神为主线，立足城乡统筹协调发展，以工业立市带动农业产业化、农村工业化城镇化，坚持“多予、少取、放活”方针，落实农村政策，改善农业发展条件，实现农民增收的根本目标。

进一步提升农业产业化水平。稳定粮食播种面积和产量，保障粮食安全。以各类示范园区为载体，扩大对台农业合作，提高“4142”绿色农产品基地整体质量，培育水果、蔬菜、水产、花卉四个优势行业和畜牧、茶叶两个重点行业。继续扶持锦溪、超大、紫山等一批农业产业龙头企业，促进农业基地向生产、加工、销运、观光等多功能综合开发发展。推进农产品标准化生产，扩展绿色食品及原产地等标志认证工作，增强应对国际市场的能力。

落实农村各项政策。深化农村税费改革，进一步落实国家、省有关取消、减免农村税负的各项规定。增加农业投入，新增财力重点向“三农”倾斜。继续稳定耕地延包制度，按“依法、自愿、有偿”的原则引导土地适度规模经营，基本完成集体林权制度改革。努力改善农村生产生活条件，继续实施农村公路“年千公里工程”和 “六千”水利工程建设，积极推进农村教育、卫生、文化、计生等工作协调发展。继续落实减负措施，切实防止农民负担反弹。

构建农业生产安全体系。严格保护耕地，确保基本农田总量不减、质量不降、用途不变。以九龙江北溪防洪工程为重点，提高江海堤防抗洪能力。完善洪水预警网络建设，增强灾害性气候测报预警能力。逐步构建动植物病虫害、森林火灾、海洋灾害、地质灾害预警信息系统，健全应急体系建设，增强防灾减灾能力。以巩固沿海防护林带为重点，加强农业生态建设，治理水土流失、青山挂白，整治农业污染。加快农产品监测网络建设，实现“餐桌污染”治理目标。

创新农村工作机制。充分发挥农村基层组织建设“三级”网络的作用，增强基层自主发展能力。探索农村“一事一议”制度化、规范化的可行方式，进一步落实村务公开，提升农村基层民主管理水平。落实农技员选聘工作，恢复、健全农技服务网络。推广“芗安”经验，培育多形式农村新型合作组织。健全登记、培训、对接、服务的一体化工作机制，推广劳务派遣制度，提高农村劳动力转移组织化程度，实现年转移6万人以上工作目标。引导农村产权制度改革，推广小额贷款“信用村”建设，改善农户生产融资条件。因地制宜引导、拓宽村级集体财力创收渠道，建立相对稳定、逐步增加的集体收入来源。

（四）创新工作机制，增强发展活力

深化国企改革要有新思路。组建市国有资产监督管理委员会，完善管理监督体制和制度体系。认真抓好最后一批列为全省战略调整企业（13家）和列入全省国企改革范围（119家）的改制工作，积极支持漳州发展、片仔癀、龙轴股份等上市公司做好资产营运，推进红旗股份上市。进一步完善力佳、糖业集团等企业的现代企业制度建设。搞活企业内部管理，健全经营、财务、分配等管理制度，加强企业经营管理人才队伍建设，提高国有企业的整体素质和综合效益。

激活民营经济要有新突破。进一步落实《加快民营经济发展的若干意见》，深化行政审批制度改革，降低企业准入门槛。加强投资引导，优化投资导向、市场、项目、科技、政策、法律等信息服务。建立健全支持中小企业发展服务体系，培育发展资金、土地、人才、技术等各类要素市场，创新扶持民营企业的融资方式。支持民营企业开展质量管理体系认证，培育优质品牌。引导民营企业制度创新、技术创新和管理创新，转变增长方式，提升整体竞争力。

提升外经贸水平要有新举措。以4·9漳台经贸恳谈会、6·18项目交易会、9·8投洽会和11·28花博会为重点平台，扩大招商成果；强化招商工作责任机制，加强招商机构、招商网络和招商队伍建设；以工业园区为载体，建立发展“飞地工业”激励机制，激发基层、社会各界招商引资的积极性；以培育壮大支柱产业为导向，加强产业链招商；以企业为主体，发挥行业协会、社团的桥梁作用，组织对口招商，开展登门招商、网上招商、中介招商，全方位拓展招商渠道；突出抓好实际到资，努力实现利用外资总量、质量有新的突破。坚持国内招商与对外招商并重，提高引进内资实效。抓住今年出口退税政策相对稳定的契机，不断优化出口商品结构、质量，增强应对国际贸易壁垒的能力，推进出口贸易总量实现新的跃升。

促进市场活跃要有新机制。加强政府政策引导，坚持市场运作为主，鼓励各类经济组织和民间资本有机结合，促进市场建设投资主体多元化。加快各类专业市场的建设和升级改造步伐，形成一批有较强区域辐射力的产品市场或交易中心，重点扶持沃尔玛商贸广场、浦口粮食市场等一批日用商品、工业产品和原辅配件、特色农产品等专业批发市场建设。组织、引导企业拓展营销，提升市场占有率，带动扩大生产规模和产业集聚。积极推广现代流通组织形式和新型营销方式，鼓励发展大型超市、连锁经营、物流配送，重点跟踪落实漳州开发区粮食物流园区、吉马物流、聚善堂药业、漳州中糖等物流配送项目建设。

（五）突出项目带动，夯实发展基础

努力提高项目工作质量。以全省产业发展布局为指导，抓紧做好“十一五”规划和港口、产业、市场、电力、交通、科技等专项规划的编制、修编工作。以规划为导向，拓宽项目开发的领域、空间和渠道。强化项目工作机制，整合项目工作机构，增加前期经费投入，加强项目开发、储备、推介、引进和落实，增强项目后续生成能力。要以积极有效的措施，提高工业项目在重点项目中的比重和大中型项目在投资项目中的比重。

积极推进重点项目建设。今年计划安排在建重点项目102个，年度投资91.5亿元，占全年固定资产投资49.9%（比去年提高4.7个百分点）。其中生产性项目60个，包含工业、能源、物流仓储等领域，年度投资62.6亿元，占重点项目年度投资计划68.4%（比去年提高16.4个百分点）。突出抓好灿坤家电扩建、福建力佳技改等重点工业系列项目，同时，积极推进招银港区深水泊位、全市电力输变网络扩容等基础设施系列项目；江滨路、西洋坪大桥等市政设施系列项目；华阳体育馆等科教文化系列项目；“六千”水利工程等防灾减灾系列项目和汽车汽配交易中心等市场网络系列项目，不断增强实现跨越式发展的支撑能力。

健全项目建设责任机制。积极应对制约项目建设的突出矛盾，在创新机制上找准推进项目工作的突破口，面向市场开发项目、融汇资金。制定《推进重点项目建设若干意见》，进一步实行项目分级挂钩责任制，建立项目信息平台和协调制度，及时协调解决项目建设中规划定点、征地拆迁、供电供水、治安管理等难点问题，为项目建设营造良好的综合环境。

（六）拓展中心城市，壮大县域经济

扩展中心城市规模，提升城市品位。全力推进新区建设，以新区重点工程和南昌东路、龙文路北段、南山景区等重大项目为带动，扩大市区规模。抓好九湖、颜厝等周边城镇的规划建设，带动南岸片区联动开发，促进中心城区沿九龙江出海口向东延伸。加强对新区建设、旧区改造的景观规划指导，培育富有闽南特色的城市风格。健全、完善城市管理体制，开展国家卫生城市、国家环保模范城市、国家园林城市创建活动，建立城市有序管理的长效机制。

努力壮大城市经济。全力推进金峰、蓝田、龙文开发区建设，着力构筑城市经济聚合的主载体。健全房地产开发用地供应制度，引导开发一批优质品牌的生态型住宅新区，促进房地产消费需求有效增长。加速发展城市运输业，合理调整运力结构，扶持龙头运输企业扩展运营，鼓励民间资本发展水陆运输，积极拓展外地客货运输市场，改善城市公共交通。整合汽车销售网络，拓展汽车消费按揭业务，降低汽车消费行政收费，促进汽车消费快速增长。巩固“中国优秀旅游城市”成果，发挥旅游资源优势，以火山地质公园为重点培育旅游品牌，以市区为中心，连接重点景区，构建具有漳州特色的滨海生态、史迹民俗、农业观光旅游网络，加强设施建设，改进旅游服务，提升旅游产业质量和效益。

推进县域经济实现梯度跃升。进一步落实鼓励县域经济发展的政策措施，扎实开展“比学赶超”活动，引导各地把优势做足，把特色做大，把实力做强，实现“三个波次”跳跃式发展。引导县城建设与开发区建设、小城镇建设有机结合，加快县域工业化、城镇化进程。加强县域间合作交流，开展基础设施、产业群体、市场体系建设等方面的分工与协作，畅通要素流动通道，促进资源互补、利益共享、联动发展。以增强财政能力为重点，努力夯实基础财源，培植新兴财源；坚持依法治税，强化税收征管；进一步调整财政支出结构，加强资金监管，切实提高资金使用效益；加强市、县资金运行调度，帮助基层乡镇缓解财政困难；建立完善县域财政增收激励机制，促进县级财政实力不断增强。

（七）坚持以人为本，促进全面发展

努力推进经济社会协调发展。继续巩固、提升教育“两基”工作成果；扎实实施中小学危房改造工程；重点推进高中阶段教育，确保当年适龄人口高中入学率达到70%；积极发展职业教育、民办教育，加快漳州高教园区、漳州师院新校区建设。积极推动文化体制改革和机制创新，加快发展公益性文化事业；突出闽南文化特色，繁荣文化创作，提供更多健康向上、群众喜闻乐见的精神文化产品。加快体育事业发展，积极开展全民健身活动，举办第十届市运会，力争在第十三届省运会取得更好成绩。加强公共卫生体系建设，健全突发性公共卫生事件应急机制，稳步推进农村卫生体制改革，组织好长泰县农村新型合作医疗体系试点工作。

促进人与自然和谐发展。实行严格的耕地保护制度，合理调整城乡建设用地结构，提高用地效益，保持耕地占补平衡；加强矿产资源管理，防止恶性、过度开采。引导发展循环经济，推动各种废旧资源的回收利用，大力倡导节约资源的绿色生活方式；进一步推进“两江两溪”等重点流域的水环境综合整治以及以三湘江为重点的市区内河专项治理；加强“创模”工作力度，力争年底前通过国家验收。认真落实计划生育责任目标，加大人口与计划生育综合改革力度，建立健全“依法管理、村（居）民自治、优质服务、政策推动、综合治理”的新机制，稳定低生育水平，提高出生人口政策符合率。

关心和改善群众生活。努力增加就业岗位，加强就业培训和指导，发展和规范职业中介服务，扩展劳动力市场建设，提升劳动者就业能力和实效。扩大养老、失业、医疗保险覆盖面，落实好下岗职工基本生活保障制度、城镇失业人员保险制度、城市和农村居民最低生活保障制度，抓好各类社会保险金的扩面、征缴、清欠和确保发放工作。切实解决“五保户”、下岗职工、城乡贫困居民、受灾群众、贫困残疾人等困难群体的生产生活问题，深入开展各项救助活动，逐步扩大城市无障碍设施配套建设，建立救助贫困群体的长效机制。

（八）推进精神文明和民主法制建设，维护社会安定稳定

努力构建平安漳州。落实综治责任制，严厉打击各种刑事犯罪和制售假烟、“六合彩”、赌博等违法犯罪活动；健全城区治安巡防体系，强化基层群防群治网络，广泛开展创建平安家园活动。切实加强信访工作，按级负责，认真解决群众反映比较集中的热点难点问题。严格实行安全生产责任制，落实以防为主方针，坚决遏制重特大事故发生。

加强精神文明建设。加快爱国主义教育基地建设，弘扬民族精神和时代精神，增强加快发展的精神动力；大力开展职业道德、社会公德和家庭美德教育，高度重视未成年人思想道德教育；深入开展文明城市（县城）、文明村镇、文明行业、文明社区等群众性精神文明创建活动，不断提高创建成效和水平；加强城乡环境卫生专项整治，营造良好的人居、创业环境。深入开展“双拥”活动，密切军政军民关系，力争我市在新一轮创建双拥模范城（县）活动中有新的突破；加强国防教育、国防动员、人民防空和民兵预备役建设，为改革、发展、稳定和国防现代化建设作出新贡献。

发扬社会主义民主。认真执行人民代表大会及其常委会的决议、决定，定期报告工作，自觉接受监督。积极支持政协参政议政，接受民主监督。完善政府配合人大的专题视察、审议制度，完善政府与政协的联席会议、对口协商制度，虚心听取各民主党派、工商联、无党派人士和人民团体的意见，广泛集中民意民智，推进决策科学化、民主化。进一步加强基层组织建设和民主建设。全面贯彻党的宗教信仰自由政策，积极引导宗教与社会主义社会相适应。

三、建设高度负责、奋发有为的人民政府

漳州发展正处在由稳步发展向跨越式发展迈进的重要转折期。必须以为民、务实、廉洁、高效为目标，不断加强执政能力建设，做到不负重托、不辱使命。

（一）突出发展，为民执政。政府的工作就是要为群众谋福利。当前，漳州群众最需要、最期盼的是发展。只有推进发展不断跃升，才能符合群众的根本意愿，才有解决问题的根本办法。要以积极的目标推进发展，一经人民代表大会确定的各项发展指标、建设项目、工作措施，必须确保落实。要扎扎实实办好市委、市政府确定的为民办实事项目，凡是向群众承诺的事项，必须坚决兑现、不打折扣。深入推进政府职能转变，努力提高为企业、为基层、为群众服务的效率和质量。要认真办理人大代表建议、政协委员提案，妥善办理群众信访，有效解决群众反映的突出问题，让群众满意、代表满意、委员满意。

（二）把握大局，科学执政。科学执政是树立和落实科学发展观的前提和保障。要坚持“五个统筹”方针，重视处理好突出发展工业与发挥农业优势的关系，扩张经济总量与依靠科技、培育人才、提升质量、增加效益的关系；推动发展提速与资源配置、生态保护、环境优化的关系。要坚持重大决策集体研究制度，完善决策咨询机制，加强政府部门与社会各界、与广大市民的沟通，广泛接纳各方建议意见，提高决策透明度与公众参与度。要进一步推行政务公开，以县级政务公开为重点，带动乡镇、村居、学校、企业、医院等机构的行政事务公开。政府的重大行政事务要在决策过程、办事进程、执行结果等方面自觉接受人大代表的法律监督、政协委员的民主监督、社会各界的群众监督和舆论监督。

（三）严于律己，依法执政。落实依法治市既要求市民要遵纪守法，更重要的是政府必须依法行政。要认真学习、执行《行政许可法》，贯彻落实《全面推进依法行政实施纲要》，提高依法行政自觉意识。一切行政行为必须严格依法办事，注意防止重实体内容、轻法定程序的倾向，努力提高依法行政的水平。要认真落实廉洁从政的各项规定，严肃查处违法违纪案件，坚决纠正部门行业不正之风，坚决纠正征用土地、房屋拆迁、企业排污、国企改制中损害群众利益，以及拖欠农民工工资等问题。政府领导、部门领导要带头从我做起，模范遵守纪律规定，严格落实廉政建设责任制，管好自己、管好家属子女、管好身边工作人员、管好分管的队伍。要进一步健全依法行政的监督机制，打造推进依法行政的良好氛围和坚实基础。强化行政监察、财务审计，从源头上建立防止滥用职权、权钱交易的有效机制。

（四）敢挑重担，争创一流。不断开创漳州发展新局面是政府的第一要务、第一责任。履行好这一职责，首先要高度负责，在保持共产党员先进性教育活动中进一步增强使命感、责任感，勇于向历史负责，向事业负责，向群众负责，奋发进取，有所作为。其次要开拓创新，不断解放思想，更新观念，在体制创新、机制创新、方式创新、科技创新上探索破解难题、推动发展的新途径。再次要求真务实，把负责精神渗透到每一项工作，每一个项目，每一条措施上，不解决问题不取得成效决不放手。同时要强化机制，加强效能监察，从机制上解决机关运行的梗塞现象，提高效率，落实责任；要把履行职责的业绩、比学赶超的动态纳入政府成员绩效考评的重点内容，激发团队精神，带领干部群众争创一流业绩，推进漳州各项事业跃上新台阶。

各位代表，我们已经走过的历程成就喜人，将要踏上的征程任重道远。让我们在市委的坚强领导下，大力弘扬爱拼敢赢、勇攀高峰的精神品格，凝心聚力，继往开来，在全面建设小康社会的历史进程中，努力创造跨越式发展的新辉煌！
